# Assessing the efficacy of two new formulations of larvicide pyriproxyfen for the control of *Aedes aegypti* using dissemination stations in two sites of Rio de Janeiro city

**DOI:** 10.1590/0074-02760200271

**Published:** 2020-10-30

**Authors:** Rosilene de Alcântara Pinto, Luiz Guilherme Soares da Rocha Bauzer, Demetrio Tederiche Borges, José Bento Pereira Lima

**Affiliations:** 1 Fundação Oswaldo Cruz-Fiocruz, Instituto Oswaldo Cruz, Laboratório de Fisiologia e Controle de Artrópodes Vetores, Rio de Janeiro, RJ, Brasil; 2 Prefeitura da Cidade do Rio de Janeiro, Secretaria Municipal de Saúde, Rio de Janeiro, RJ, Brasil; 3 Instituto de Biologia do Exército, Laboratório de Entomologia, Rio de Janeiro, RJ, Brasil

**Keywords:** Aedes aegypti, pyriproxyfen, vector control, dissemination stations

## Abstract

**BACKGROUND:**

*Aedes aegypti* is the primary transmitter of several arbovirus with great impact in human health. Controlling vector mosquitoes is an essential and complex task. One promising control method is to use mosquitoes as a vehicle to disseminate tiny particles of juvenile-killing insecticides, such as pyriproxyfen (PPF), to breeding sites.

**OBJECTIVES:**

We aimed to investigate the capacity of *Ae. aegypti* to disseminate two new formulations of PPF in two sites of Rio de Janeiro city for assessment of the efficacy of these products.

**METHODS:**

Dissemination stations impregnated with powder and liquid new formulations of PPF were installed in two test sites. Ovitraps were used in the test sites and in a control site for monitoring the presence of *Ae. aegypti* throughout eggs collection.

**FINDINGS:**

Entomological indices indicated that the new formulations of PPF were efficient in reducing eggs abundance. Liquid formulation performed better than powder formulation. Ready-to-use formulations of PPF can be quickly applied in the field and can be replaced after a few months.

**MAIN CONCLUSIONS:**

New formulations of PPF associated with mosquito dissemination approach make a valuable vector control strategy, managing to cover places of difficult access for whatever reason. New formulations application requires less labour, being economically attractive.

*Aedes aegypti* (Linnaeus, 1762) is a dipteran, belonging to the Culicidae family, found in tropical and subtropical zones between latitudes 35°N and 35°S. This species has an urban, anthropophilic and synanthropic habitat, coexisting with humans in their homes. *Ae. aegypti* has historically been the primary transmitter of urban yellow fever, dengue fever, chikungunya and Zika fever viruses, which have had the greatest impact on human health.^1^ It is a species that exhibits skip oviposition whose females visit several breeding sites to lay a few eggs in each.^2^ ^,^ ^3^ *Ae. aegypti* females lay eggs just above the water line, preferably in artificial and transitory containers located in the home or yard, such as buckets, drums, tires, vases, among others.^4^

Effective vaccines exist only for yellow fever. New vaccines are being developed for dengue fever and Zika fever, but they still show limitations.^5^ Due to the lack of effective vaccines to dengue, Zika and chikungunya, other approaches are essential to control the spread of these pathogens, such as vector control. However, controlling vector mosquitoes is a complex and dynamic task that relies on many actions that logically add to each other.^6^ The control tactics usually focus on insecticide spraying to kill adult mosquitoes in combination with the identification and elimination of breeding sites.^7^ ^,^ ^8^ However, insecticide spraying has only transient effects on adult mosquito and can be seriously jeopardised by insecticide resistance. As for the elimination of breeding sites, the coverage is often so low, what makes control campaigns largely ineffective.^8^

It is possible to increase breeding-site coverage by using adult mosquitoes that show a skip oviposition behaviour as a vehicle to disseminate tiny particles of juvenile-killing insecticides to breeding sites.^9^ ^-^ ^11^ In this approach, mosquitoes are lured to dissemination stations containing larvicides particles that adhere to the insect’s body and are later transferred to clean breeding sites subsequently visited for oviposition. Pyriproxyfen (PPF) is classified as a juvenile hormone analog and serves as a potential larvicide that can safely be used even in drinking water.^12^ This larvicide kills immature mosquitoes at extremely low doses and has been successfully used in several studies using the autodissemination approach for mosquito control.^9^ ^-^ ^11^ ^,^ ^13^ ^,^ ^14^ In Rio de Janeiro city, Brazil, this approach may come to complement the routine actions of vector control programs that require dwellings visit, as there is a large number of closed, abandoned, empty and rejected properties throughout the city’s area. Also, it is worth noting that regions most vulnerable to *Ae. aegypti* are also susceptible to urban violence in Rio de Janeiro. These communities commonly experience some level of social marginalisation, which impacts on public health policy and community health workers duties,^15^ such as elimination of breeding sites and preventive treatment of water deposits.

In the present work, we investigated the capacity of *Ae. aegypti* to disseminate two new formulations of PPF (liquid and powder) in two sites of Rio de Janeiro city, demonstrating the true potential that this methodology can have in vector control.

## MATERIALS AND METHODS

*Dissemination stations -* Stations measuring 17 cm height and 19.5 cm diameter, with capacity for 3,000 mL of tap water, were installed for PPF dissemination. The stations were lined inside with a thin porous TNT - non-woven black fabric, cut in the ideal size for covering the inner wall of the trap. The fabric was previously washed to remove the waterproofing, making it an ideal environment for oviposition.

Liquid EW PPF (1 mL) (Sumilarv, Sumitomo, London, UK) was added inside 500 dissemination stations and 0.5 g of concentrated powder PPF (Sumilarv, Sumitomo, London, UK) was added inside other 500 dissemination stations. Later, 3,000 mL of water was added in all containers. The new PPF formulations were added directly in the water placed inside the dissemination stations, requiring no differentiated procedure for impregnating the fabric. Just mixing the powder or liquid with water was enough to impregnate the fabric. The dissemination stations were installed in one third of the dwellings located in the listed blocks of the trial sites, that is, one in three houses. Dissemination stations deployment took place from November 2017 to November 2018. The monitoring of the dwellings with the disseminating stations was carried out monthly by the management agents. The fabric of some traps was replaced during the project, according to the agent evaluation. The powder product was replaced after four months from the beginning of the experiment and the liquid formulation was replaced after six months from the beginning of the experiment. 

*Traps for monitoring (ovitraps) -* For the monitoring of *Ae. aegypti* and the verification of positivity and eggs density, ovitraps were used as an ovipositional attractant for females.^16^ ^,^ ^17^ Ovitraps provide useful data on the spatiotemporal distribution of mosquitoes because this monitoring allows one to check the presence and density of the vector at a local site.^18^ ^,^ ^19^ Ovitraps were 600 mL cups measuring 9.5 cm height and 12 cm diameter baited with a 0.04% yeast solution. One millilitre from a yeast stock solution (6 g yeast plus 50 mL H_2_O) was diluted in 300 mL tap water inside the ovitrap. A wooden paddle (12 cm × 3 cm) was clamped inside the ovitrap, having the rough side facing the inner part of the cup, serving as a substrate for oviposition.

The ovitraps were distributed in such a way as to cover the entire area where​ ​dissemination stations were installed. The distribution was one ovitrap every 10 dissemination stations. For the control area, ovitraps where installed every 150 m. The ovitraps were georeferenced for mapping. They were installed in October 2017 (month 1), three weeks before the dissemination stations installation. The paddles were changed weekly for verification of positivity and density. After removal of the dissemination stations in November 2018 (month 14), the ovitraps were kept in the studies sites for three weeks in December 2018 (month 15), for verification of positivity and density without direct interference from the larvicide due to dissemination. Two months after the removal of the dissemination stations, in February 2019 (month 17), the ovitraps were installed again in the same places to monitor the positivity and density.

**Fig. 1:**
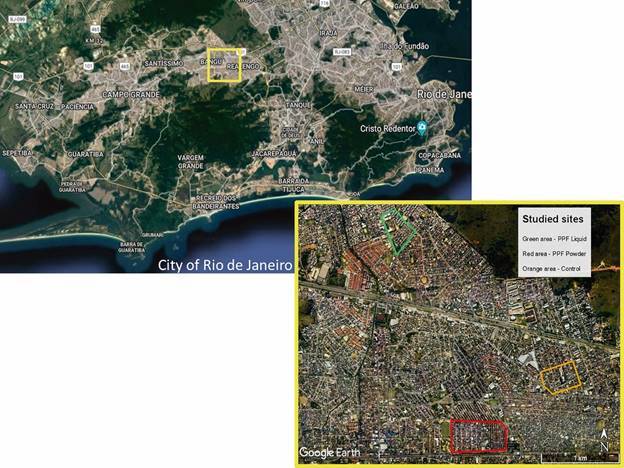
map of Rio de Janeiro city, Brazil, indicating the studied region and closer aerial view of the studied sites.

*Study areas -* The experiments were carried out in three sites located in a highly urbanised area in the western part of Rio de Janeiro city. Fig. 1 shows aerial views of the studied sites available from Google Earth Pro software (version 7.3.37699, Google, Mountain View, USA). The chosen sites are lower middle-class residential areas with houses with a small yard. These areas were chosen because they have similar demographic and geographic characteristics and are among the neighbourhoods with the highest number of dengue cases in Rio de Janeiro city in 2017. The sites are approximately 2 km apart.

The liquid formulation of PPF was tested in the site of Jardim Bangu. In this area, we installed 500 dissemination stations (one in three dwellings) and 50 ovitraps (one in 30 dwellings). The powder formulation of PPF was tested in the site of Bangu/Padre Miguel. In this area, we also installed 500 dissemination stations (one in three dwellings) and 50 ovitraps (one in 30 dwellings). The site of Realengo was considered as the control area. In this area, we installed only ovitraps (one in 30 dwellings). Dissemination stations were not installed in Realengo. 

*Entomological indices computation and spatial and statistics analysis -* Entomological indices were calculated for the infestation measurement. They indicated the presence and abundance of *Ae. aegypti* females throughout the amount of eggs, as well as the periods in which reproduction varies.

The Ovitraps Positivity Index (OPI) shows the mosquitoes’ spatial distribution in the studied area. It is the ratio between the number of positive traps and the number of traps examined multiplied by one hundred.^20^ The Eggs Density Index (EDI) indicates the vector’s population abundance. It is the ratio between the number of eggs and the number of positive ovitraps.^21^ The Eggs Average Index (EAI) indicates the average amount of eggs deposited per paddle. It is the ratio between the number of eggs and the number of traps examined.^22^ Kolmogorov-Smirnov test was used to verify the assumption of normality of the entomological indices described above. After these initial tests, the non-parametric Mann-Whitney *U* test was performed to compare monthly values of OPI, EDI and EAI among the three sites evaluated. Comparisons showing p-values < 0.05 were considered significantly different. The statistical software SPSS 23.0 (IBM SPSS Statistics for Windows, Armonk, USA) was used for analysis assistance. 

The ovitraps were labelled by GPS and the data was transferred to QGIS 3.4 program.^23^ The heat maps (Kernel) were prepared using the QGIS 3.4 program based on the monthly quantity of eggs collected in the georeferenced ovitraps during the study period. The colour variation is the representation of the monthly EDI in each of the study areas and control area. This variation is due to the amount of eggs in the ovitraps in addition to the proximity of the traps to each other. 

*Ethics -* Dwellings owners who agreed to have their homes used to install the disseminating stations received a summary of the project for knowledge and signed a consent form, authorising the stations to remain in their dwellings for a period of 13 months. In the control area of Realengo, dwellings owners who agreed to have their homes used to install the ovitraps also received a summary of the project for knowledge and signed a consent form. In the areas where PPF was tested, dwelling owners who agreed to have their homes used to install the ovitraps received a summary of the project for knowledge. 

## RESULTS

*Entomological indices -* The high OPI values observed indicate that mosquitoes were evenly distributed in all areas during the entire period of the study (Table I). The liquid formulation of PPF was shown to be more effective in restricting the distribution of *Ae. aegypti* than the powder formulation. This is because of OPI values of Jardim Bangu were significantly smaller than OPI values of the control area during eight of the 13 months of PPF dissemination (month 4 and month 8 up to month 14). For the PPF powder formulation, used in Bangu/Padre Miguel, OPI values were significantly smaller than OPI of the control area only for the months 11 and 14. After the removal of the dissemination stations, in month 14, it was observed that OPI values no longer differed significantly among the studied areas, indicating the success of PPF liquid formulation in reducing the spatial distribution of *Ae. aegypti* eggs*.*

**TABLE I:** Ovitraps Positivity Index (OPI) mean, standard deviation (SD) and Mann-Whitney *U* test for the pairwise comparisons among the areas treated with pyriproxyfen (PPF) liquid formulation (L), PPF powder formulation (P) and control (C)

	P	L	C	P x L	L x C	P x C
Month_Year	Mean % (SD)	Mean % (SD)	Mean % (SD)	p-value	p-value	p-value
1 Oct_17	80.3 (13.7)	62.3 (10.9)	64.1 (5.6)	0.275	0.827	0.275
2 Nov_17	85.6 (6.6)	69.8 (8.8)	82.2 (8.4)	0.083	0.059	0.773
3 Dec_17	89.2 (7.4)	79.9 (7.6)	90.2 (6.7)	0.083	0.108	1.000
4 Jan_18	85.8 (6.6)	78.2 (4.0)	93.4 (3.2)	0.076	0.016	0.917
5 Feb_18	91.7 (8.9)	81.4 (9.0)	93.4 (3.2)	0.439	0.121	1.000
6 Mar_18	89.8 (2.7)	89.3 (5.6)	91.3 (2.6)	0.663	0.885	0.468
7 Apr_18	84.8 (6.7)	81.2 (4.7)	90.3 (6.4)	0.375	0.081	0.149
8 Mai_18	81.5 (7.0)	73.5 (7.9)	86.8 (4.7)	0.173	0.009	0.249
9 Jun_18	87.1 (10.2)	70.7 (8.7)	91.8 (10.0)	0.043	0.042	0.384
10 Jul_18	91.4 (4.9)	71.7 (4.0)	96.3 (3.3)	0.009	0.009	0.093
11 Aug_18	87.5 (3.0)	80.3 (5.3)	92.1 (1.2)	0.081	0.021	0.020
12 Sep_18	86.4 (5.7)	77.4 (5.5)	91.2 (3.4)	0.059	0.021	0.309
13 Oct_18	93.1 (1.8)	82.1 (5.5)	94.3 (4.4)	0.009	0.028	0.598
14 Nov_18	86.3 (1.9)	79.8 (4.8)	95.0 (4.7)	0.083	0.021	0.029
15 Dec_18	93.8 (3.1)	89.8 (2.9)	94.0 (5.6)	0.439	0.439	1.000
17 Feb_19	86.7 (1.4)	80.6 (4.3)	92 (5.7)	0.121	0.121	0.121

Significant p-values are in bold.

The EDI values computed indicated a reduction in *Ae. aegypti* eggs abundance both in the area treated with liquid PPF and in the area treated with powder PPF, when compared with the control area (Table II). For the liquid formulation, the eggs abundance reduction was significant in nine of the 13 months analysed, as observed in Table II. As for the powder formulation, the mosquito eggs abundance reduction was significant in seven of the 13 months analysed, as observed in Table II. After the removal of the dissemination stations in month 14, it was observed that EDI values no longer differed significantly among the studied areas, indicating the success of both liquid and powder formulation of PPF in reducing the abundance of *Ae. aegypti* eggs*.*

**TABLE II:** Eggs Density Index (EDI) mean, standard deviation (SD) and Mann-Whitney *U* test for the pairwise comparisons among the areas treated with pyriproxyfen (PPF) liquid formulation (L), PPF powder formulation (P) and control (C)

	P	L	C	P x L	L x C	P x C
Month_Year	Mean (SD)	Mean (SD)	Mean (SD)	p-value	p-value	p-value
1 Oct_17	134.5 (69.5)	111.2 (19.4)	62.5 (17.4)	0.513	0.050	0.275
2 Nov_17	222.5 (41.7)	135.6 (18.7)	109.3 (19.5)	0.021	0.149	0.021
3 Dec_17	233.6 (30.4)	130.6 (18.7)	197.0 (21.6)	0.021	0.021	0.149
4 Jan_18	136.6 (21.4)	115.1 (31.1)	119.2 (25.4)	0.465	0.754	0.347
5 Feb_18	156.1 (50.6)	132.5 (30.6)	131.6 (77.5)	0.439	1.000	0.439
6 Mar_18	151.7 (9.6)	100.2 (13.0)	168.1 (19.0)	0.021	0.021	0.248
7 Apr_18	131.8 (21.2)	77.5 (31.5)	169.3 (16.9)	0.043	0.021	0.043
8 Mai_18	131.1 (28.3)	87.6 (16.1)	195.6 (45.0)	0.009	0.009	0.028
9 Jun_18	159.8 (34.1)	92.2 (31.6)	216.5 (20.4)	0.021	0.021	0.043
10 Jul_18	113.7 (25.2)	83.5 (9.1)	162.3 (40.8)	0.076	0.009	0.117
11 Aug_18	103.1 (18.8)	76.2 (9.1)	154.6 (29.6)	0.043	0.021	0.021
12 Sep_18	96.2 (11.8)	85.6 (2.3)	188.4 (39.3)	0.248	0.021	0.021
13 Oct_18	108.6 (20.0)	84.6 (11.4)	171.6 (35.1)	0.028	0.009	0.028
14 Nov_18	103.8 (19.3)	81.8 (6.2)	146.3 (7.8)	0.083	0.021	0.021
15 Dec_18	124.0 (55.5)	99.5 (8.4)	142.1 (15.8)	1.000	0.121	1.000
17 Feb_19	98.4 (53.7)	95.8 (26.0)	97.6 (39.9)	1.000	1.000	1.000

Significant p-values are in bold.

The EAI values computed indicated a reduction in *Ae. aegypti* eggs average both in the area treated with liquid PPF and in the area treated with powder PPF when compared with the control area (Table III). For the liquid formulation, the eggs average reduction was significant in nine of the 13 months analysed, as observed in Table III. As for the powder formulation, the eggs average reduction was significant in seven of the 13 months analysed, as observed in Table III. After the removal of the dissemination stations in month 14, it was observed that EAI values no longer differed significantly among the studied areas, indicating the success of both liquid and powder formulations of PPF in reducing the *Ae. aegypti* eggs average.

**TABLE III:** Eggs Average Index (EAI) I mean. standard deviation (SD) and Mann-Whitney *U* test for the pairwise comparisons among the areas treated with pyriproxyfen (PPF) liquid formulation (L). PPF powder formulation (P) and control (C)

	P	L	C	P x L	L x C	P x C
Month Year	Mean (SD)	Mean (SD)	Mean (SD)	p-value	p-value	p-value
1 Oct_17	114.3 (71.4)	69.0 (16.1)	40.6 (14.2)	0.513	0.077	0.275
2 Nov_17	189.3 (29.7)	95.9 (25.0)	90.1 (18.7)	0.021	1.000	0.021
3 Dec_17	210.0 (42.7)	103.5 (10.1)	178.4 (30.4)	0.021	0.021	0.149
4 Jan_18	116.4 (12.9)	90.5 (26.9)	102.9 (19.4)	0.076	0.251	0.347
5 Feb_18	145.4 (60.3)	109.2 (36.9)	124.2 (76.6)	0.439	1.000	0.439
6 Mar_18	136.3 (11.5)	88.9 (5.6)	153.7 (20.6)	0.021	0.021	0.564
7 Apr_18	111.2 (14.8)	63.2 (26.6)	153.0 (19.4)	0.043	0.021	0.043
8 Mai_18	105.9 (17.3)	64.9 (16.7)	169.8 (42.0)	0.009	0.009	0.009
9 Jun_18	123.1 (27.4)	58.4 (19.1)	185.6 (50.2)	0.021	0.021	0.083
10 Jul_18	104.7 (27.6)	59.7 (5.8)	156.3 (40.4)	0.009	0.009	0.047
11 Aug_18	90.3 (17.4)	61.3 (9.1)	142.4 (27.2)	0.021	0.021	0.021
12 Sep_18	83.2 (12.3)	66.2 (4.6)	171.5 (34.1)	0.021	0.021	0.021
13 Oct_18	101.1 (18.2)	69.5 (11.1)	162.1 (36.3)	0.016	0.009	0.028
14 Nov_18	89.8 (17.8)	65.1 (4.4)	139.1 (12.4)	0.043	0.021	0.021
15 Dec_18	117.2 (55.9)	89.4 (10.4)	133.0 (6.9)	1.000	0.121	1.000
17 Feb_19	85.0 (45.1)	77.8 (25.1)	91.0 (42.2)	1.000	0.439	0.439

Significant p-values are in bold.

**Fig. 2:**
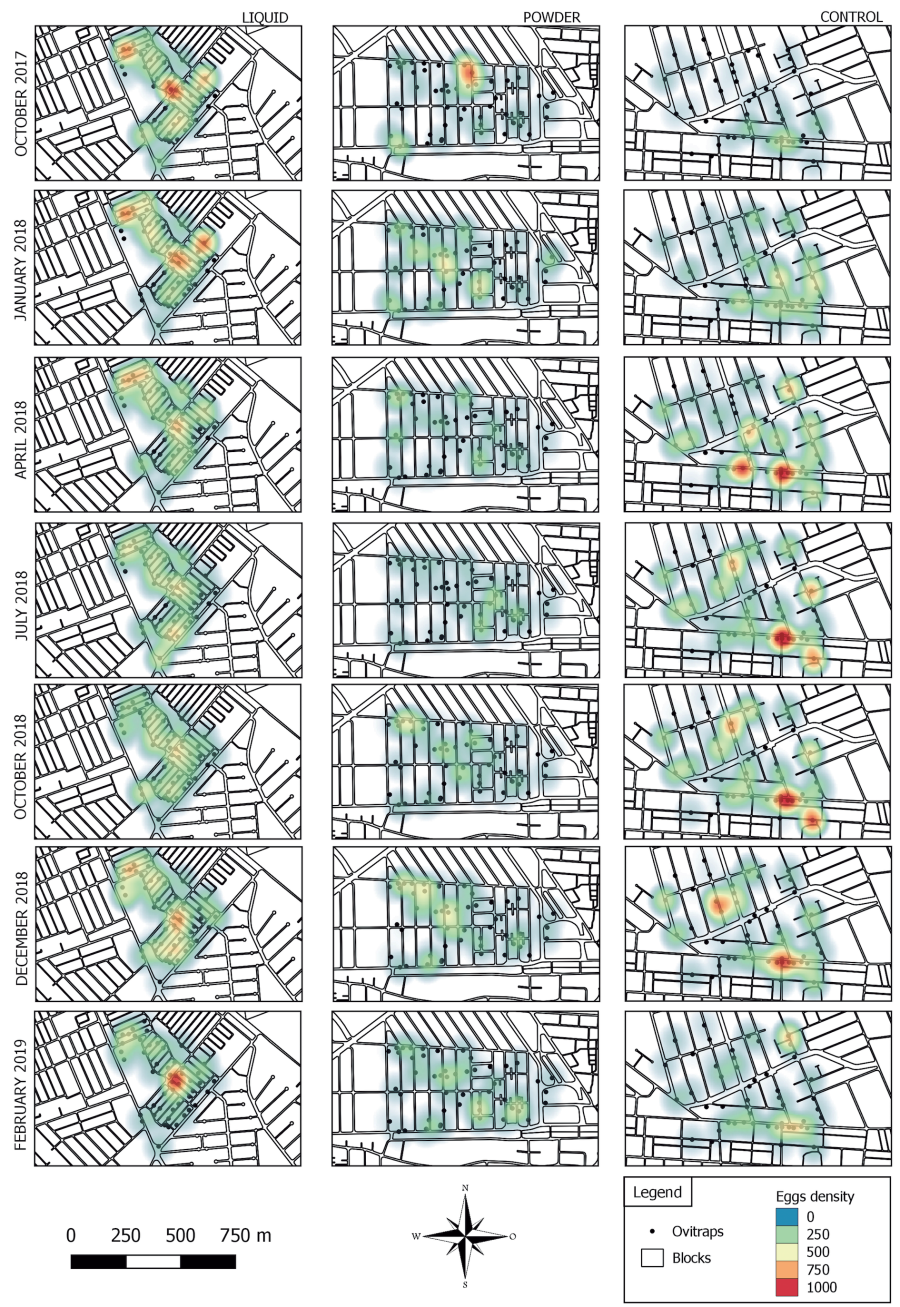
Kernel heat maps for the studied sites.

*Spatial analysis -* Fig. 2 shows the Kernel heat maps. In some months, hotspots were observed, showing a greater amount of eggs both in the dissemination stations and in the ovitraps distribution space, being represented by the red colour on the maps. In the beginning of the experiments (October 2017, month 1), the area of Jardim Bangu had the highest density of eggs. In April 2018 (month 7), it was possible to observe a strong reduction in the density of eggs in Jardim Bangu due to the effects of PPF liquid formulation. From month 7 up to October 2018 (month 13) the highest densities of eggs were observed in the control area, indicating the effects of both liquid and powder formulations of PPF in reducing the density of *Ae. aegypti* eggs. In the last two months (December 2018 and February 2019), after the removal of the dissemination stations, the density of eggs showed a rapid increase in Jardim Bangu, with eggs density values even higher than the control area in the last month. This result indicates that liquid formulation was successful in reducing eggs density in Jardim Bangu and that the density rapidly recovery to the original level after removal of the dissemination stations. 

## DISCUSSION

Vector control in Brazil is mainly based on the elimination of breeding sites by endemic agents by visiting house to house.^24^ This approach has not been very efficient because it is not being carried out as recommended by the vector control program, which recommends six annual cycles with coverage for at least 90% of homes. In this way, the implementation of tools that reduce the number of visits and the number of agents is essential to advance in the control of *Ae. aegypti*. In urban areas, such as Rio de Janeiro city, there are some factors that prevent a high coverage of inspected houses, such as the high number of closed or abandoned houses and the increase of violence in poor areas. In this context, the impregnation of dissemination stations with different formulations of insecticide using the mosquitoes as a vehicle to disseminate insecticide to potential breeding sites is a good solution. Autodissemination or horizontal transfer of PPF results in a reduction of mosquito population by juvenile mortality and subsequent recruitment of adults. Studies showed that autodissemination occurs and successfully increases the mortality rate of larvae that are exposed (reviewed in Hustedt et al.^25^). 

Devine et al.^10^ distributed 1 L plastic pots lined with damp black cloth dusted with pulverised PPF granules in the field in Iquitos, Peru. The authors achieved overall reductions in adult emergence of 42-98% thus achieving high coverage of aquatic mosquito habitats. Sentinel aquatic habitats were distributed throughout the experimental site and their attendant larvae were closely monitored to determine how much PPF dissemination was occurring. This is a convenient approach to measure impact, adopted by all subsequent studies. However, this is also problematic, as it is not known whether artificial habitats reflect mortality in natural habitats or how they affect adult mosquito density. Abad-Franch et al.^13^ used these autodissemination stations in a field trial in Manaus city, northern region of Brazil. The authors found greater than 10-fold rise in juvenile mosquito mortality. They also found greater than a 10-fold decrease in adult mosquito emergence. Caputo et al.^11^ ^)^ used the autodissemination strategy in Rome, Italy, this time mediated by *Aedes albopictus*. The use of 0.5% powder caused more than 50% mortality in artificial habitats. However, there was no attempt to demonstrate amplification between exposure sites and aquatic habitats as the number of dissemination sites was equal to the number of artificial habitats. 

In previous studies using dissemination stations and PPF, Sumilarv 0.5G was ground to a powder to dust the fabric used inside the stations.^10^ ^,^ ^11^ ^,^ ^13^ ^,^ ^14^ Some authors commented on the need for a better formulation of PPF, as time was wasted having to grind the granules to acquire the consistency of powder.^14^ The ready-to-use formulations without the need for gridding represent a great gain of time for the application of the technique directly in the field, in addition to no need for specialised labour to prepare and apply the product, thus reducing costs for extra training. The present study was innovative as ready-to-use formulations were used to impregnate the dissemination stations. In previous studies using PPF in dissemination stations, the fabric lining the inner wall of the stations was dusted only once and the monitoring lasted just a few days.^10^ ^,^ ^11^ PPF dissemination stations have already been used for long term monitoring which lasted four to five months.^13^ ^,^ ^14^ In this case, dissemination stations were placed in sheltered locations and checked fortnightly throughout the months of the trial to refill water and re-dust fabric with PPF using a brush. Here we showed that PPF new formulations can be quickly applied in the water and can be replaced less frequently, as they lasted longer. Both the way of impregnation and the interval of reapplication of the products are of great importance for control programs, as they are directly related with the number of people necessary to apply the products. 

Ovitraps were used to monitor the studied sites in order to assess positivity and eggs density. Ovitraps are recommended as a method of secondary investigation in areas not infested by the vector and are used as efficient surveillance tools, as they have high sensitivity for detecting the presence of the vector and can be used for defining control strategies.^26^ Compared with larval surveys, ovitraps were shown to be more sensitive and cost effective at low mosquito densities.^27^ ^,^ ^28^ Compared with adult traps, ovitraps were shown to be the ones with best sensitivity as they never presented null indices.^29^ Although not measuring directly the adult population, ovitraps were shown to capture adult variation very well, with strong association with climate and consistently following the adult mosquitoes pattern detected by adult traps.^29^

Our results show that ovitraps positivity was always very high, with little difference among the sites evaluated. The observed positivity indices indicate wide spatial distribution of *Ae. aegypti* eggs*.* Although OPI values were high for all sites studied, it was possible to observe a small effect of PPF dissemination in reducing ovitraps positivity for both liquid and powder formulations. The liquid formulation performed better than the powder formulation, as indicated by the higher number of months liquid OPI values were significantly smaller than control OPI values. It is important to mention that after the dissemination stations had been removed from the studied sites, OPI values were no longer significantly different in the pairwise comparisons among the sites. This indicates that without PPF dissemination, the spatial distribution of *Ae. aegypti* eggs no longer suffer a small reduction. Despite the high positivity, eggs density decreased in the sites where dissemination stations were installed, due to the success of PPF dissemination by mosquitoes. This is indicated by both EDI and EAI values, which were significantly higher for both liquid, and powder formulations in the majority of months evaluated when compared with the control. Liquid formulation also performed better to reduce density when compared with powder formulation. This is indicated by the higher number of months both EDI and EAI liquid formulation values were significantly different from the control when compared with the number of months both EDI and EAI powder formulation values were significantly different from the control. 

Like for OPI values, as soon as the dissemination stations were removed from the studied sites, both EDI and EAI values were no longer significantly different in the pairwise comparisons among the sites. This indicates that without PPF dissemination, the density of *Ae. aegypti* eggs no longer suffer a strong reduction. Also, this indicates that the recovery to the original density levels is very rapid. In the control area of Realengo, both positivity and eggs density remained high, indicating that without the effects of PPF dissemination these indices remain the same reflecting no seasonal variation. It is possible that there was migration of mosquitoes from nearby places to the studied sites keeping the ovitraps positive, as already observed in a previous study.^13^

PPF powder and liquid new formulations have proven to be effective in decreasing the density of mosquito eggs and can become a complementary strategy in controlling *Ae. aegypti*. The reduction was observed in all monitored dwellings around the installed dissemination stations, indicating that inaccessible houses could also have been treated with PPF. This is of great importance especially if one considers particularities of highly urbanised areas such as Rio de Janeiro city where violence and other factors prevent a high coverage of dwellings inspected by health agents.^15^ This study indicates the possibility to accomplish the PPF treatment in unverified breeding sites and properties not surveyed using the mosquito itself as a larvicide disperser, managing to reach places of difficult access for whatever reason. 

There are some limitations in the autodissemination methodology, especially in urban areas. Field trials suggest that autodisseminated PPF can increase juvenile mosquito mortality and reduce adult mosquito emergence. However, the effect tends to reduce over time. Also, as observed in this study, the reduction is low enough that additional tools may need to be used in combination with PPF to reduce *Aedes* populations to zero.^25^ Another point is that urban topography will impact significantly on adult mosquito flight, which can be constrained. It can directly affect the PPF dispersal capacity. Urban heterogeneity needs to be considered when optimising the design of autodissemination trials.^30^ Future studies should look further at defining standardised approach for application of PPF, optimum design of devices and distribution in highly urbanised areas. 
